# Microbial biostimulant reshapes carbon and nitrogen metabolism in olive trees: dendrochronological insights into enhanced growth and climate adaptation

**DOI:** 10.3389/fpls.2026.1805787

**Published:** 2026-05-12

**Authors:** Silvia Portarena, Nicola Cinosi, Mona Mazeh, Daniela Farinelli, Paola Pollegioni, Chiara Traini, Rodrigo Jose de Vargas, Fabiola Villa, Enrico Brugnoli, Syed Wasif Ahmed, Paolo Cherubini, Franco Famiani

**Affiliations:** 1 Institute of Research on Terrestrial Ecosystems (IRET), National Research Council (CNR), Porano, Italy; 2 National Biodiversity Future Center, Palermo, Italy; 3 Department of Agricultural, Food and Environmental Sciences (DSA3), University of Perugia, Perugia, Italy; 4 State University of Western Paraná (Unioeste), Marechal Cândido Rondon, Brazil; 5 Forest and Soil Ecology, Swiss Federal Institute for Forest, Snow and Landscape Research WSL, Birmensdorf, Switzerland; 6 Faculty of Forestry, University of British Columbia, Vancouver, BC, Canada

**Keywords:** carbon allocation, dendrochronology, microbial biostimulants, nitrogen acquisition, *Olea europaea* L., stable isotopes, sustainable agriculture

## Abstract

Microbial biostimulants are increasingly proposed as sustainable tools to enhance crop performance and resilience under climate change. However, their ecophysiological effects and underlying mechanisms remain insufficiently understood, particularly in woody perennial species. This study investigated the effects of MICOSAT F^®^ microbial biostimulant on growth and ecophysiological traits of two-year-old *Olea europaea* L. cv. Leccino plants under controlled greenhouse conditions. For the first time, we integrated agronomic measurements with dendrochronological analyses and intra-annual assessments of carbon and nitrogen concentration (C%, N%) and their stable isotope composition (δ¹³C, δ¹^5^N) in tree rings. Biostimulant-treated trees exhibited significantly larger stem diameter, height, lateral branching, and total biomass compared with controls. Treated trees showed lower stem wood C%, suggesting increased C allocation to non-structural carbohydrates and belowground symbionts. Furthermore, treated trees displayed significantly depleted δ¹³C values with reduced interannual variation, indicating enhanced stomatal conductance and more stable photosynthetic discrimination. Depleted δ¹^5^N signatures reflected a shift toward microbially-mediated N acquisition pathways rather than increased absolute N availability. These findings demonstrate that MICOSAT F^®^ biostimulant fundamentally alters plant C and N metabolism, promoting growth while enhancing physiological stability - key traits for potential climate resilience in sustainable olive cultivation systems.

## 1 Introduction

The olive tree (*Olea europaea* L.), an evergreen species of the *Oleaceae* family, is among the oldest cultivated crops in the Mediterranean basin. Its nutritional benefits, derived from a balanced fatty acid profile and high antioxidant content (Anselmi et al., 2022), have driven a steady rise in global consumption and the intensification of cultivation systems.

Global climate change represents a critical challenge to modern agriculture, affecting ecosystem functioning, crop productivity, and food security. Rising temperatures, altered precipitation regimes, and extreme events are already exerting significant pressure on agricultural systems worldwide (Vinci et al., 2023). Olive trees, although drought-tolerant, are increasingly exposed to climatic conditions that exceed their adaptive capacity, particularly in Mediterranean environments characterized by water scarcity and soil degradation (Miserere et al., 2021; Gavrichkova et al., 2022). Climate change also strongly affects soil functions by enhancing erosion rates through high-intensity rainfall events and altering organic carbon (C) availability and nutrient cycling because of changes in soil moisture and temperature regimes. In olive-growing areas, these impacts are often exacerbated by conventional management practices, including intensive tillage, high fertilizer and pesticide inputs and mechanized harvesting, all of which contribute to environmental degradation (Daimonakos et al., 2026).

At the same time, the increasing global demand for olive oil has promoted the spread of intensive and high-density olive production systems (Cinosi et al., 2025). While these systems enhance short-term productivity, they are often associated with high water requirements, reduced soil quality, and increased vulnerability to climatic stress. Consequently, a transition toward sustainable, resource-efficient, and climate-resilient practices is essential (Bedbabis et al., 2014; Andrade et al., 2010; Di Vaio et al., 2021).

Among emerging strategies to enhance plant performance under both biotic and abiotic stress conditions, the use of biostimulants has gained increasing attention as a promising tool for sustainable agriculture (Almadi et al., 2020; Castiglione et al., 2021; Ritter et al., 2025). Biostimulants are defined as natural substances and/or microorganisms that stimulate plant physiological processes improving nutrient use efficiency, growth, yield, and stress tolerance, independently of their nutrient content (Du Jardin, 2015; Brown and Saa, 2015; Mazeh et al, 2021; Franzoni et al., 2022; Dias et al., 2022). Their positive effects on plant growth have been reported in a wide range of crops, including cereals, horticultural species, and fruit trees (Luciani et al., 2019; Alì et al., 2021; Azeem et al., 2022; Melloni and Cardoso, 2023; Abdelmoaty et al., 2022). Among these, microbial biostimulants, represent a key category within this group, as they encompass living microorganisms, including arbuscular mycorrhizal fungi (AMF) and Plant Growth-Promoting Bacteria (PGPB), capable of establishing beneficial associations with host plants (Adedayo and Babalola, 2023; Kaushal et al., 2023).

In European olive cultivars, root-associated microbial communities constitute a reservoir of such beneficial microbes (Fernández-González et al., 2019; Dias et al., 2024a). Direct root inoculation with AMF or PGBP, either isolated from olive orchards or included in commercial mixtures, mostly composed of fungi *Glomus* sp., *Funneliformis* sp. and *Rhizophagus* sp, or bacteria such *Bacillus*, *Pseudomonas*, *Azospirillum*, and *Rhizobium*, has been proved to enhance nutrient uptake, growth and tolerance to abiotic stresses such as drought, salinity, and low temperature (Bizos et al., 2020; Dias et al., 2024a; El-Shazly and Ghieth, 2019; Hassena et al., 2021; Galicia-Campos et al., 2022; Calvo‐Polanco et al., 2016; Ouledali et al., 2018; Aganchich et al., 2022; Dias et al., 2024b; Chialva et al., 2021).

In this context, MICOSAT F^®^ (Quart, Aosta, Italy) is a microbial consortium containing AMF, saprophytic fungi, and rhizosphere bacteria, capable of establishing mutualistic associations with plant roots, enhancing nutrient and water uptake, improving soil structure, and stimulating the synthesis of plant secondary metabolites (Du Jardin, 2015; Luciani et al., 2019; Rai et al., 2021; Fusco et al., 2022; Melini et al., 2023). Notably, MICOSAT F^®^ has already shown effectiveness in reducing the incidence of Olive Quick Decline Syndrome (OQDS), associated with *Xylella fastidiosa* subsp. *pauca* (Giovannetti et al., 2019). However, despite the growing interest in MICOSAT F^®^ biostimulant, its application in olive cultivation remains partially unexplored, particularly from an ecophysiological perspective.

This knowledge gap is largely due to the complexity of biostimulant formulations, which makes it challenging to elucidate their modes of action and their interactions with plant physiological processes (Salvioli di Fossalunga and Bonfante, 2023; Arinaitwe et al., 2025). Dendroecological approaches provide a powerful framework for investigating tree growth dynamics and physiological responses to environmental conditions and management practices (Fonti et al., 2010; Cherubini et al., 2021; Portarena et al., 2025; 2026). Tree-ring width variations reflect adaptive growth responses to environmental constraints and resource availability (De Micco et al., 2019). Moreover, the analysis of carbon concentration (C%) and stable isotope composition (δ¹³C) in tree rings provides retrospective insights into photosynthetic performance, stomatal regulation, and intrinsic water-use efficiency (Monson et al., 2018; Portarena et al., 2024).

Similarly, tree-ring nitrogen concentration (N%) and stable isotope composition (δ¹^5^N) reflect the isotopic signature of available N sources and offer valuable information on N uptake pathways and cycling within plant–soil systems (Gerhart and McLauchlan, 2014; Gessler et al., 2014; van der Sleen et al., 2017). Yet despite their potential, these proxies remain underexploited in dendrochronological research (Reimchen and Arbellay, 2018).

The present study aims to evaluate the effects of the microbial biostimulant MICOSAT F^®^ on growth and ecophysiological traits of two-year-old *Olea europaea* L. cv. Leccino plants grown under controlled pot conditions. The Leccino cultivar was selected due to its documented tolerance to *Xylella fastidiosa* (Sabella et al., 2018; La Notte et al., 2024). For the first time, we combined, agronomic measurements with dendrochronological analyses and intra-annual assessments of C and N concentration, as well as their isotopic composition (δ¹³C and δ¹^5^N) in tree rings. This integrated approach allowed us to investigate how microbial biostimulant application modulates stem growth, C assimilation, and water- and nitrogen-use efficiency in young olive trees, providing novel ecophysiological insights into the potential role of biostimulants in sustainable olive cultivation.

## 2 Materials and methods

### 2.1 Sample treatment, cultivar, plant growth measurements and AMF propagule quantification

The study was conducted between 2022 and 2024 in Central Italy (Umbria region), at the experimental laboratory of the Department of Agricultural, Food and Environmental Sciences of the University of Perugia, located in Deruta (PG, Italy) (42° 58’ 18” N, 12° 24’ 11” E).

The experiment was performed in a greenhouse on 20 one-year-old potted olive trees, cultivar Leccino, 35–40 cm tall (at the beginning of the trial). The Leccino cultivar was selected as it is one of the most widely cultivated olive cultivars in Italy. It is native to central Italy, and is characterized by high vigor and good adaptability to different environmental conditions. It produces medium-sized fruits with moderate to high oil content and is particularly valued for its regular bearing. Moreover, Leccino has a high tolerance to *Xylella fastidiosa*, and also for this reason it is one of the most used cultivars in new plantations (Sabella et al., 2018).

**Figure 1:**
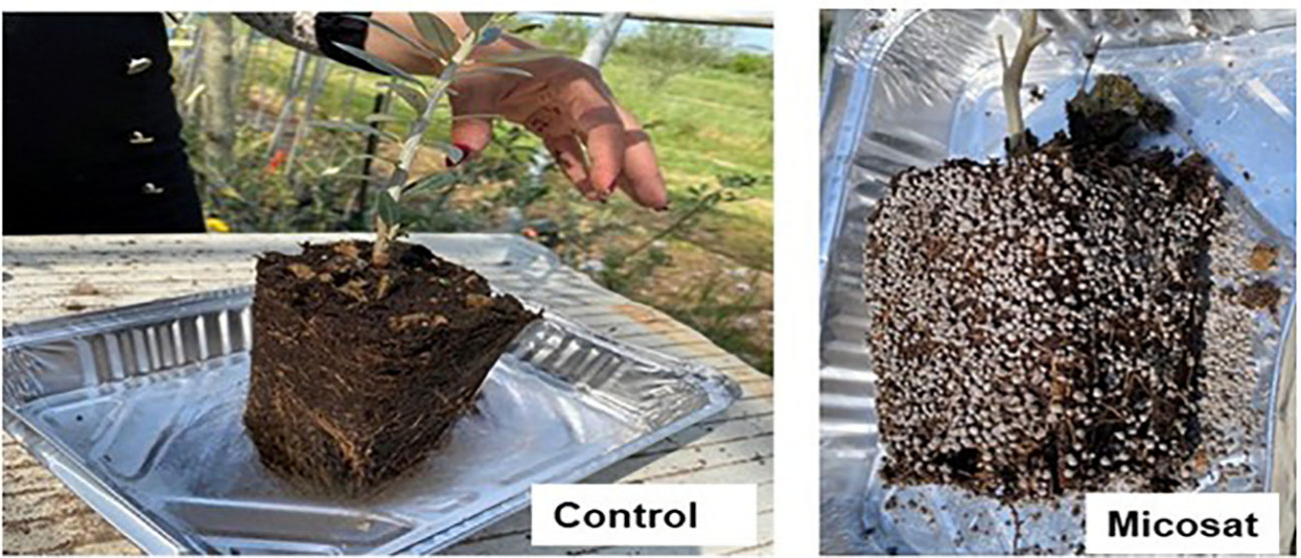
Application of the microbial biostimulant around the tree substrate at the time of transplanting.

In 2022, at the beginning of the trial, at transplanting (May) into a larger pot (2.4-litre pots) using a peat and pozzolan substrate, the microbial biostimulant (MICOSAT F^®^, C.C.S. Aosta s.r.l., Quart – AO, Italy) was applied at a rate of 21 g/plant, distributed around the substrates of transplanted plants, while untreated plants were used as controls (**Figure 1**). Subsequently, the pots were fertilized with 1.8 g of Nitrogen (Urea 46%).

In 2023, the microbial biostimulant was re-applied to the treated plants in April (12 g/plant), July (4 g/plant) and September (4 g/plant), for a total of 20 g/plant. In each of the three periods, the biostimulant was dispersed in water and poured onto the substrate. Furthermore, in May, all plants were fertilized with 3.0 g of Nitrogen (Urea 46%). Peat was used since it is generally sterile, usually pest and pathogen-free, stable in structure, with a consistent pH and a consistent and low nutrient content. While pozzolan, that is a volcanic substrate made of natural rock, even if it is not sterile by default, was chosen since it is essentially inert and low in organic matter, which gives it some similar advantages to sterile substrates (Litterick et al., 2025).

MICOSAT F^®^ is a consortium containing spores and mycelium of arbuscular mycorrhizal fungi (AMF) of the genus *Glomus* (*Glomus* spp.GB 67, *Glomus mosseae* GP 11, *Glomus viscosum* GC 4), saprophytic fungi (*Trichoderma harzianum* TH 01, *Pochonia chlamydosporia* PC 50), rhizosphere bacteria (*Agrobacterium radiobacter* AR 39, *Bacillus subtilis* BA 41, *Streptomyces* spp. SB 14) and a yeast (*Pichia pastoris* PP59).

All plants were maintained with a standard daily irrigation regime using a drip irrigation system.

From the beginning to the end of the experiment, plant growth was monitored by measuring stem diameter, plant height, and the number and length of primary, secondary and tertiary branches.

At the end of 2023, four plants per treatment were destructively sampled to determine the biomass (dry weight) of their various components (roots, stem, branches and leaves) and to assess the concentration of AMF propagules.

After confirming root colonization using methyl blue staining, the concentration of AMF propagules in the rhizospheric soil was estimated using the Most Probable Number method, as described by Wang et al. (2008). Results were expressed as the number of infective propagules per 100 gram of dry soil.

Relative air humidity and temperature data were obtained from a meteorological station adjacent to the experimental site (WatchDog^®^ Wireless ET Station, 3G/HSPA).

### 2.2 Tree-ring sample collection and preparation

Eight olive trees, collected from that used for growth measurements, 4 control and 4 treated by biostimulant (height: 1.84 ± 0.45 m; stem diameter: 1.20 ± 0.11 cm; mean ± standard deviation) were used for wood sampling. One disk was collected from the stem of each tree at 5 cm above ground level at the end of June 2024. Disks were air-dried and polished using progressively finer sandpaper to enhance cell visibility and delineate tree-ring boundaries.

### 2.3 Tree-ring width analysis

Ring width (RW) was measured at a resolution of 0.01 mm using a LINTAB system (Rinntech, Heidelberg, Germany) coupled with a Leica MS5 stereoscope (Leica Microsystems, Wetzlar, Germany) and TSAPW in software (Frank Rinn, Heidelberg, Germany). Due to asymmetric cambial growth, RW was measured along four radii per disk, and the four series were averaged after visual cross-dating using common marker years and patterns (Stokes and Smiley, 1968).

Cross-dating accuracy and measurement consistency were validated with the dplR package (Bunn, 2008) in R.

### 2.4 Tree ring sectioning and C, N, δ¹³C, and δ^15^N analyses

Tangential slices (~15 μm thick) were prepared using a sliding microtome (GSL1, WSL). These slices spanned the period from the onset of earlywood (EW) formation in 2022 to July 2024. Each slice was cataloged according to its corresponding growth ring and its position within the EW or latewood (LW).

Analyses of the ^13^C/^12^C and ^15^N/^14^/N isotope ratios were performed using an isotope ratio mass spectrometer (isoprime precision, Elementar UK Ltd, Manchester, UK) connected to an elemental analyzer (PYROcube (Elementar Analysensysteme GmbH, Langenselbold, Germany). An aliquot of 0.5 mg wood dry matter was collected to determine the concentration and isotope composition of carbon and nitrogen (C%, N%, δ^13^C, δ^15^N) The isotopic compositions were scale-normalized with the IAEA international standards and are expressed as ‰ notation according to Equation 1:

(1) $$\delta \;\left(‰\right)\;=\;\left(R_{s}-\;R_{std}\right)/R_{std}\times \;1000$$

where R_s_ is the isotope ratio of the sample and R_std_ is the isotope ratio of the international standard.

L-glutamic acid USGS 40 glutamic acid, NBS-22 fuel oil and IAEA-CH6 sucrose were used for the scale normalization of the measured δ¹³C values to the VPDB international standard (Portarena et al., 2024).

IAEA-600 caffeine, USGS40 glutamic acid and IAEA-NO-3 KNO3 were used for the scale normalization of the measured δ^15^N values to the IAEA international standard (Gavrichkova et al., 2022).

The standard deviation (SD) of replicate measurements for both δ¹³C and δ^15^N standards was 0.1‰.

#### 2.4.1 Standardization of the C, N, δ¹³C, and δ^15^N time series

Annual variation in tree-ring width resulted in differing numbers of C%, N%, δ¹³C,and δ^15^N measurements per year across samples. To allow comparisons, the number of observations was standardized on a yearly basis by using, for each earlywood and latewood fraction, the tree with the fewest measurements as a reference. For trees with a higher number of sections, adjacent measurements were proportionally averaged to match the number of observations of the reference tree, to create uniform time series with the same number of points across trees. While this procedure allows direct comparison among samples, it may attenuate fine-scale intra-annual signals in trees with more sections.

### 2.5 Statistical analysis

Ring width (RW) data were converted into basal area increment (BAI) calculated as the difference between the basal area of two consecutive years (Equation 2):

(2) $$BAI_{n}=\;\pi \;(r^{2}n_{–}\;r^{2}n_{\;-\;1})$$

where r_n_ and r_n-1_ represent the cumulative ring radius at the end of year of formation n and the previous year respectively.

For RW, BAI, C%, N%, C/N, δ¹³C and δ^15^N, mean values and standard errors (SE) were calculated based on four plants per treatment group (control and biostimulant-treated). For growth parameters SE were calculated from 20 plants per treatment group.

Data normality was tested with the Shapiro-Wilk test. The One-way ANOVA was performed to evaluate differences in organ-specific biomass and the concentration of AMF propagule (assessed at the end of second growing season). When significant effects were detected, pairwise comparisons among treatment means were performed using Fisher’s least significant difference (LSD) test.

Repeated measures ANOVA was applied to assess the effects of interannual variability (YEAR, random effect), treatment (TR, fixed effect), and their interaction on stem diameter, height growth, lateral branches, RW and BAI. Sum of squares (SS), mean square (MS), and F-values were calculated.

Linear mixed-effects models (LMMs) were used to test the effect of biostimulant treatment on wood chemical and isotopic traits (C (%), N (%), C/N ratio, δ^13^C, and δ^15^N). Treatment was specified as a fixed effect, while individual tree identity was included as a random effect to account for the non-independence of repeated observations within trees (The basic LMM structure used was: outcome ∼ treatment + (1|individual tree)). For all analyses statistical significance was set at p< 0.05.

All statistical analyses were performed using R (R Development Core Team 2021, Vienna, Austria).

## 3 Results

### 3.1 Weather conditions

**Figure 2:**
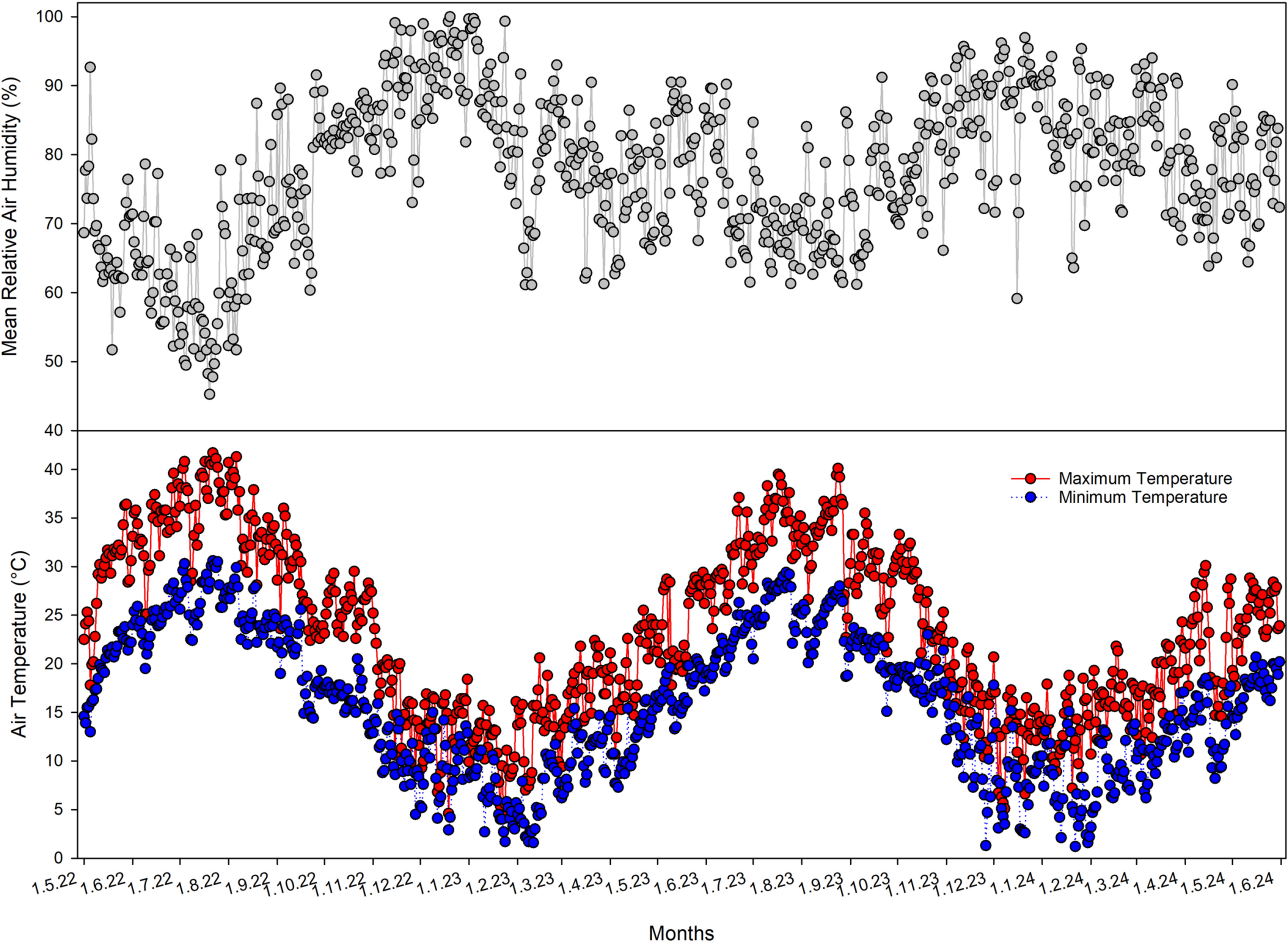
Daily relative air humidity and maximum and minimum temperature obtained from a meteorological station adjacent to the experimental site.

**Figure 2** showed the temporal dynamics of air relative humidity (RH) and air temperature from May 2022 to May 2024.

Both the daily relative air humidity (RH) and air temperature (T) exhibited a seasonal pattern, with RH showing higher values during autumn–winter and lower values during late spring–summer and T maximum (max) values during summer months and minimum in winter. The warmest conditions occurred in July for both 2022 and 2023, with Tmax reaching approximately 41.7 °C in 2022, and 40.1 °C in 2023.

### 3.2 Vegetative growth and AMF propagule quantification

The repeated measures ANOVA indicated a significant effect of year and treatment on stem diameter, tree height and length of lateral branches/shoots (**Table 1**).

**Table 1:** Repeated measures ANOVA for stem diameter, height growth and length of lateral branches across years (random effect) and treatment (fixed effect).

Effect	Stem diameter				Tree height				Length of later branches			
	SS	MS	F	p	SS	MS	F	p	SS	MS	F	p
Year	1217	608	1432	0.000	458744	229372	1363	0.000	1814662	907331	51	0.000
Treatment	11	11	26	0.000	10166	10166	60	0.000	82714	82714	5	0.033
Year*Treament	4	2	5	0.004	4111	2055	12	0.000	44118	22059	1	0.293

Significant p-values (< 0.05) are shown in red. The symbol * indicates the interaction between the effects (year × treatment).

**Figure 3:**
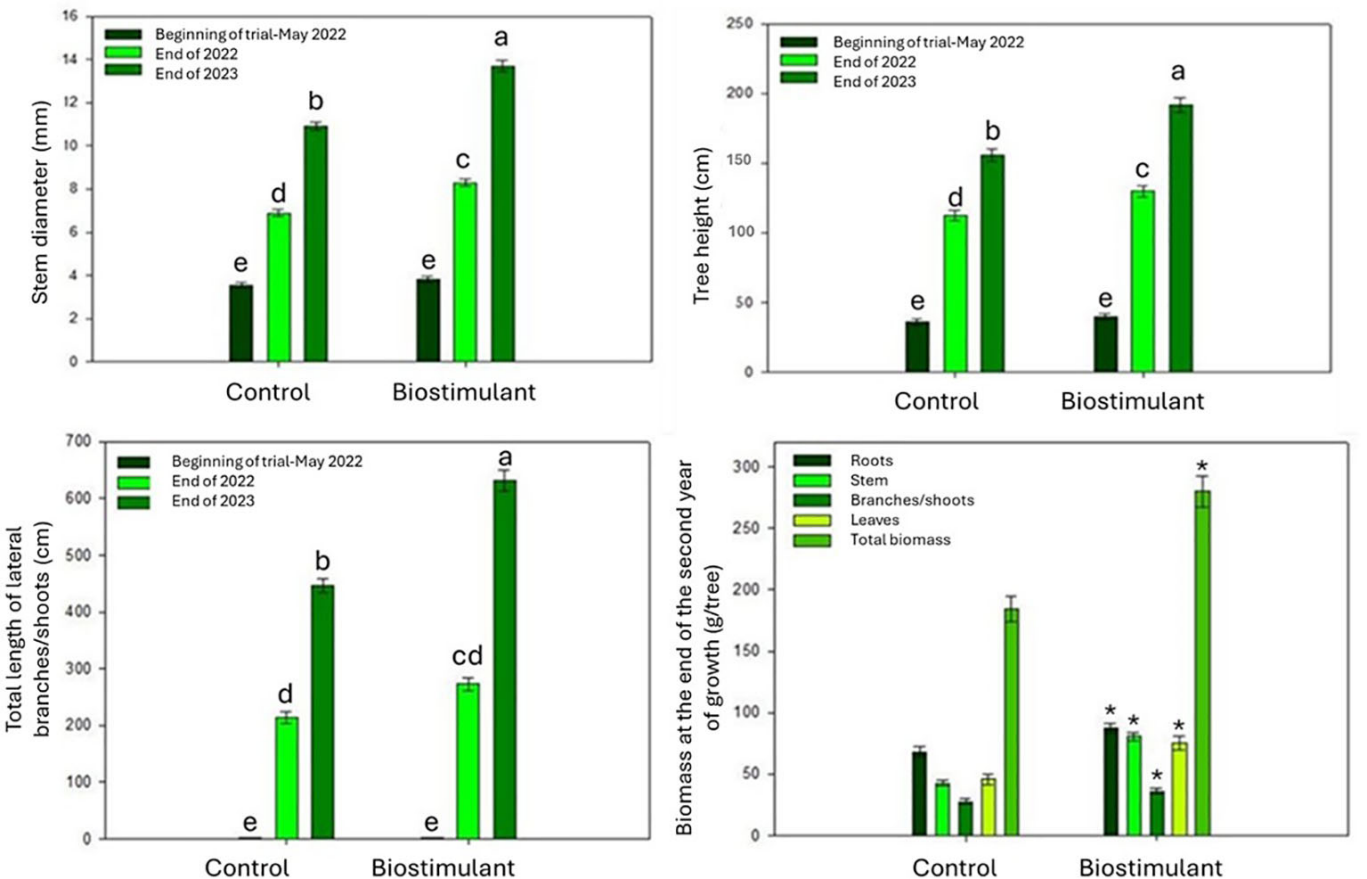
Two-year effects of the biostimulant on stem diameter, height growth, lateral branches, and organ-specific biomass measured at the end of 2023. Bars are SE; different letters indicate p ≤ 0.05.

In addition, treatment had a significant effect on cumulative biomass, expressed as the dry weight of roots, stem, leaves, and branches, at the end of the 2023, second growing season (**Figure 3**).

Plants treated with the biostimulant showed significantly greater stem diameter and height growth compared with control plants over the two-year period.

The biostimulant treatment also significantly increased the number and length of lateral branches arising from the stem at the end of second growing season (2023) whereas no significant differences were detected at the end of the 2022 growing season.

Total dry biomass was significantly higher in treated plants at the end of 2023, with increases observed across all plant components: roots, stem, branches, and leaves.

Quantification of arbuscular mycorrhizal fungi (AMF) propagules in the rhizosphere showed significantly higher density of propagules in plants treated with MICOSAT F^®^ than in untreated controls. Treated plants reached 2.02 × 10³ propagules per 100 g dry soil, compared to 1.46 × 10³ propagules per 100 g dry soil in the control, representing a 38% increase.

### 3.3 Tree-ring widths analyses

The repeated measures ANOVA indicated a significant effect of year and type of tree on both RW and BAI (**Table 2**).

**Table 2:** Repeated measures ANOVA for ring width (RW) and basal area increment (BAI) across years (random effect) and treatment (fixed effect). .

Effect	RW				BAI			
	SS	MS	F	p	SS	MS	F	p
Year	2.204	0.735	23.578	0.000	5284.689	1761.563	83.569	0.000
Treatment	0.252	0.252	8.092	0.012	278.988	278.988	13.235	0.002
Year*Treatment	0.298	0.099	3.190	0.052	129.288	43.096	2.044	0.148

Significant p-values (< 0.05) are shown in red. The symbol * indicates the interaction between the effects (year × treatment).

**Figure 4:**
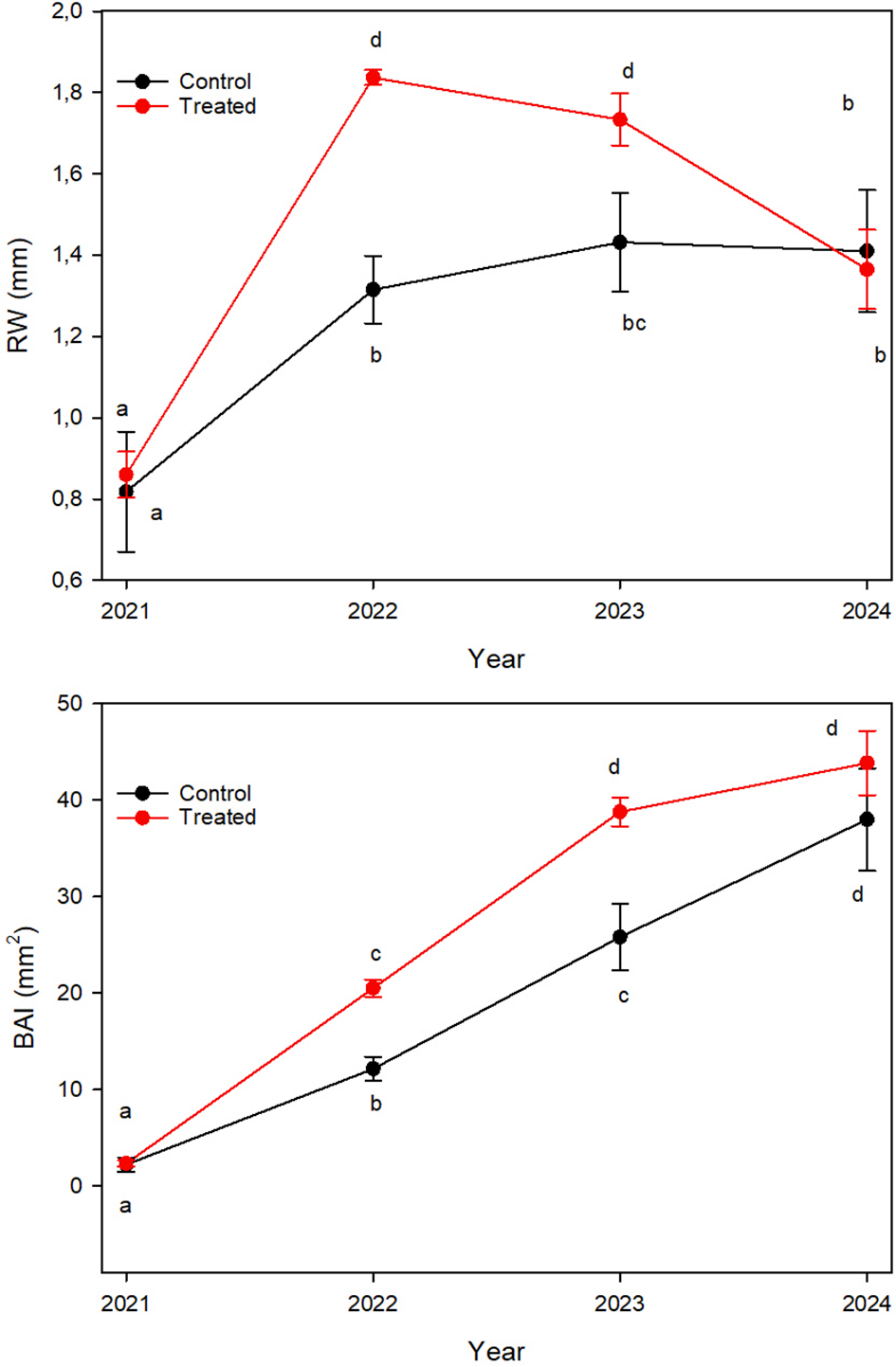
Component of radial growth in cv. Leccino: above the inter-annual RW, ring width; below the BAI, basal area increment. The values are expressed as mean ± SE. Statistical differences were assessed using Fisher’s *post hoc* multiple comparison test. Different letters indicate statistically significant differences at p< 0.05.

The stems of the sampled trees exhibited growth increases throughout the study period, from 2022 to June 2024 (**Figure 4**).

A strong correlation was observed between the RW of treated and control plants (R = 0.91, P< 0.001). Treated plants showed notable increases in both parameters, highlighting the positive impact of the biostimulant application on growth performance.

By late June 2024, when stem discs were collected, differences in RW between treated and control trees were no longer statistically significant. Although BAI remained higher in treated plants, the magnitude of the growth advantage had diminished, suggesting a gradual convergence in radial growth rates between treatments toward the end of the study period.

### 3.4 Intra-annual C%, N%, C/N, δ¹³C, and δ^15^N analyses and their correlations

**Figure 5:**
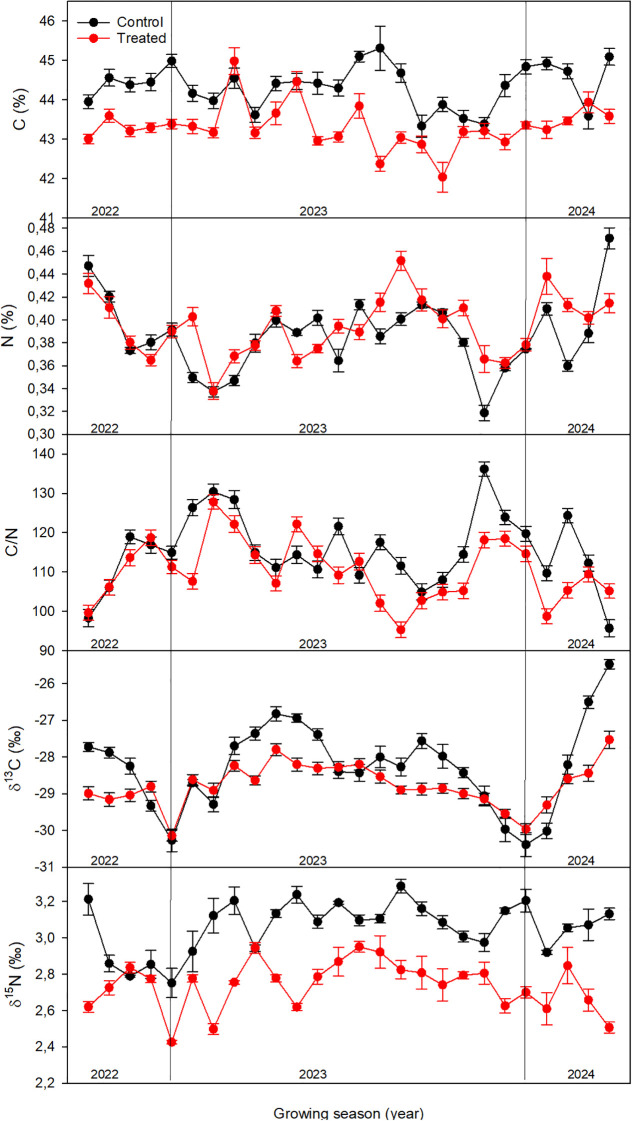
Intra-annual temporal dynamics of wood C%, N%, C/N, δ¹³C and δ^15^N across tree rings of *Olea europaea* L. (cv. Leccino). Treatments are represented by different colors: red symbols for biostimulant-treated trees, black symbols for control trees. The values are reported as mean values ± SE, standard errors.

The intra-annual temporal dynamics of wood C%, N%, C/N, δ¹³C and δ^15^N revealed distinct physiological responses of biostimulant-treated and control Leccino olive trees (**Figure 5**).

LMM results showed a significant (p<0.05) effect of biostimulant treatment on C%, C/N, δ¹³C and δ^15^N (**Table 3**).

**Table 3:** Linear mixed-effects models testing the effect of biostimulant treatment on intra-annual wood elemental concentrations (C%, N%), elemental ratios (C/N), and stable isotope compositions (δ^13^C, δ^15^N). .

Response variable	Effect type	Parameter	Estimate (β)/std. dev. (σ)	SE/residual σ	p-value
C%	Fixed	Treatment	β = −1.03	SE = 0.113	<0.001
	Random	ID	σ = 0.374	σ = 0.816	–
N%	Fixed	Treatment	β = 0.007	SE = 0.005	0.153
	Random	ID	σ = 0.008	σ = 0.039	–
C/N	Fixed	Treatment	β = −4.33	SE = 1.598	0.007
	Random	ID	σ = 1.039	σ = 11.53	–
δ^13^C	Fixed	Treatment	β = −0.520	SE = 0.144	<0.001
	Random	ID	σ = 0.239	σ = 2.1346	–
δ^15^N	Fixed	Treatment	β = −0.617	SE = 0.2960	0.038
	Random	ID	σ = 0.239	σ = 2.1346	–

Treatment was included as a fixed effect and tree identity (ID) as a random effect. Degrees of freedom DF = 203. Significant p-values (< 0.05) are shown in red.

Despite the limited temporal variability, control trees maintained higher C% (ranging between 43.3 and 45.3%) than biostimulant-treated trees (ranging between 42 and 43.3%). The seasonal pattern in control trees during 2023 showed increasing C% values during early earlywood (EW) formation followed by progressive C% depletion during latewood (LW) stem development. In contrast, treated trees exhibited more irregular intra-annual fluctuations with lower mean C%. Despite non-significant differences between treatments (p = 0.153), both groups displayed a seasonal pattern with N% increase during early-middle ring formation phases, followed by progressive decline toward later wood development stages.

Treated trees exhibited significantly lower C/N ratios than controls.

Treated trees showed more depleted δ¹^3^C values compared with controls. However, similar fluctuations were observed with values increasing in earlywood (EW) and decreasing in latewood (LW). The most negative δ¹³C values occurred near the transition between LW and EW, where annual ring formation likely commenced, while the highest δ¹³C enrichments were found mid-ring, between EW and LW (Portarena et al., 2025).

Treated plants exhibited a lower overall interannual variation (2.8‰; SD = 1.3‰) than controls (3.9‰; SD = 1.8‰).

Moreover, treated trees showed significantly more depleted δ¹^5^N values compared with controls. δ¹^5^N values ranged between 2.8‰ and 3.3‰ in control trees and between 2.4‰ and 2.9‰ in treated trees.

At the intra-annual scale, δ¹^5^N displayed a slight seasonal pattern in both treatments, characterized by a phase of increase and then decrease during wood formation. Notably, in treated trees, δ¹^5^N reached a minimum during mid-season, and subsequently increased again toward the later stages of wood development.

**Figure 6:**
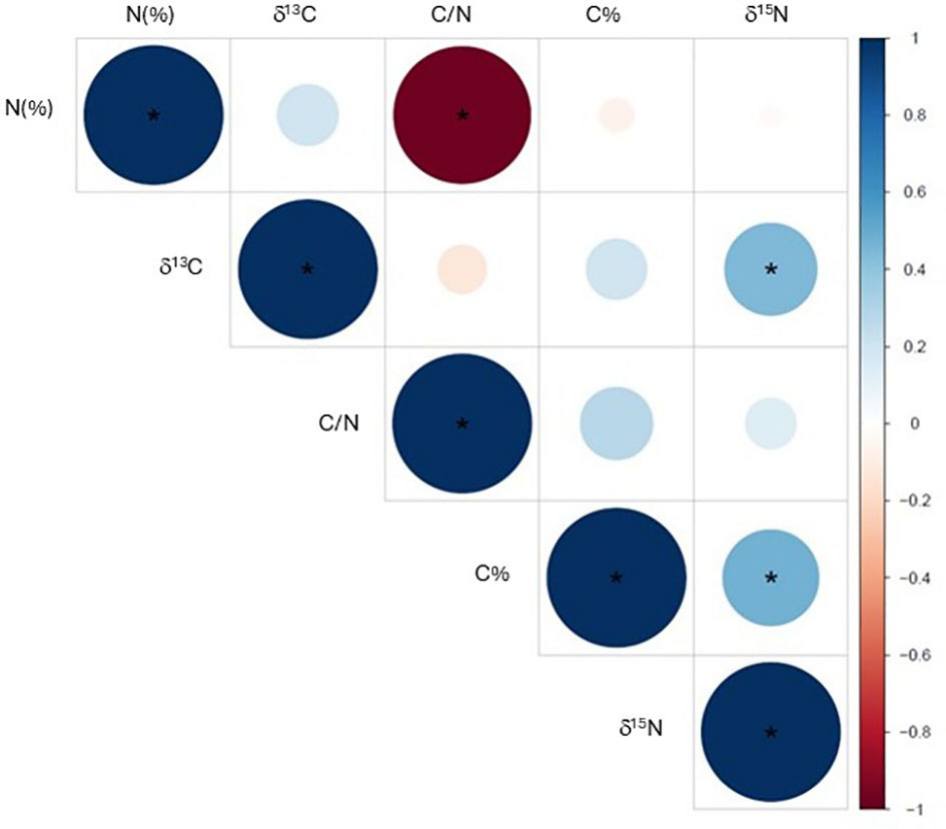
Correlation heatmap showing pairwise relationships among N%, δ¹³C, C/N, C%, and δ¹^5^N. Circle size and color intensity are proportional to the magnitude of the Pearson correlation coefficient. Blue indicates positive correlations and red negative correlations. The symbol * indicates correlations with p< 0.05.

**Figure 6** presents the bivariate correlations between elemental concentrations (C%, N%), elemental ratios (C/N), and stable isotope compositions (δ^13^C, δ^15^N) for both control and biostimulant-treated trees. Pearson correlation analysis was conducted on all data points combined from both treatments across the 2022–2024 period. As the precise timing of formation of the intra−annual wood slices within the growing season could not be determined, it was not possible to associate them with specific climate variables; therefore, climate-based correlation analyses were not performed.

No significant correlations were observed among the studied variable with the exception of the C%-δ^15^N and δ^13^C-δ^15^N relationships. The significant correlation between C/N and N is a mathematical artifact arising from the fact that C/N is computed directly from N variable.

## 4 Discussion

Olive is a thermophilous Mediterranean species well adapted to hot and dry summers (Cherubini et al., 2003; Sofo et al., 2008). However, extreme summer maximum temperatures can impose significant physiological constraints. Optimal photosynthetic performance in olive is generally observed at temperatures around 25–30 °C, while T_max_ values exceeding 35 °C are frequently associated with partial stomatal closure, reduced CO_2_ assimilation, and growth (Angelopoulos et al., 1996; Miserere et al., 2021).

Stem diameter, height, and lateral branching as well as the total biomass and its components (root, stem, branches/shoot, and leaf biomass) all increased significantly in treated trees over the two-year study period. These gains in growth performance likely reflected the combined action of the biostimulant’s microbial consortium: AMF (*Glomus* spp. *GB 67, G. mosseae GP 11, G. viscosum GC 41*), and biocontrol agents such as rhizosphere bacteria, *Agrobacterium radiobacter, Bacillus subtilis, Streptomyces* spp., and saprophytic fungi, *Trichoderma harzianum, Pochonia chlamydosporia* (Bizos et al., 2020; Dias et al., 2024a). The olive root-associated microbioma (both endosphere and rhizosphere) is cultivar-genotype dependent, influenced by environmental, pedological, and agronomic conditions (Fernández-González et al., 2019). However, Chialva et al. (2021) attested that root-microbial assembly of Leccino cultivar is highly stable across seasons in Italy and includes a reservoir of beneficial microbes promoting plant-growth such as AMF-*Glomus, Dominikia* and *Rhizophagus*. According to Seifi et al. (2014), in the mutual symbiosis with young olive plants, *Glomus mosseae* also in combination with other *Glomus* species, favored the increment of shoot and root biomass, and mineral nutrition. The increase in C% observed in control trees during the early phase of ring formation in 2023 was consistent with enhanced photosynthetic carbon (C) supply and then deposition of C-rich structural compounds, such as cellulose and lignin, during active earlywood formation. This phase was typically followed by a relative decline during latewood development, when foliar sinks progressively divert C to support late-season physiological processes. In treated trees, the more irregular intra-annual C% pattern, together with overall lower values, may reflect a shift in C partitioning during xylem development. One possible explanation is that the biostimulant-associated microbiome increased belowground C demand, either through stimulation of root growth or direct C transfer to symbiotic microorganisms (Lin and Jones, 2022; Adedayo and Babalola, 2023). In line with Lekberg et al. (2013) and Mekahlia et al. (2013), the C flux from plants to AMF may persist across seasons as a symbiotic cost, while remaining tightly coupled to plant C assimilation and nutrient acquisition benefits. Although the exact proportion of C allocated by olive trees to AM mycelium has not yet been established, broader evidence indicates that plants may transfer between 3% to 20% of net primary production to mycorrhizal fungi, with woody species allocating approximately 3% of assimilated C to AMF mycelium (Hawkins et al., 2023). The persistent C demand may contribute to shifts in plant C partitioning strategies, favoring greater allocation to non-structural carbohydrates (NSC) within woody tissues to meet the metabolic requirements of symbiotic associations (Hartmann and Trumbore, 2016). Given that NSC have a lower C mass fraction than structural compounds such as cellulose and lignin, an increased relative contribution of NSC would be expected to reduce the overall C% of the wood matrix. However, in the absence of direct measurements of NSC, this interpretation remains speculative, and alternative explanations cannot be excluded. Biostimulant-induced microbial consortia may also trigger broader metabolic adjustments in the host plant, including changes in C assimilation efficiency, secondary metabolism, and wood biochemical composition, such as lignin-to-cellulose ratios. Consistently, inoculation with *Bacillus subtilis* and *Trichoderma harzianum* has been shown to enhance nutrient availability, antibacterial activity (Bouaichi et al., 2019), and phenolic and antioxidant metabolism (Graziani et al., 2022) of olive trees. Collectively, these findings suggest that overall metabolic rearrangements induced by the biostimulant microbial consortium, in combination with enhanced belowground C sinks, may contribute to the modulation of wood C% in treated olive trees.

The partial recovery of C% observed in early 2024, coinciding with the absence of biostimulant applications after September 2023, supports this integrated interpretation. As microbial-driven belowground C demand declined, a greater proportion of assimilated C may have been redirected toward above-ground structural tissues (Luciani et al., 2019). This shift is consistent with the concurrent convergence of stem radial growth between treated and control trees observed by mid-2024 (**Figure 4**) suggesting a short-term effect of MICOSAT F^®^.

This trend may be explained by (i) root-bound constraints limiting the expansion of the symbiotic network and (ii) the transient contribution of additional microorganisms in the consortium (such as *Trichoderma harzianum* and plant growth‐promoting rhizobacteria such as *Bacillus subtilis*) whose effects may decline over time without repeated inoculation, even when AMF colonization persists (Qin et al., 2022).

The δ¹³C data provided insights into photosynthetic performance and stomatal regulation. Ouledali et al. (2018) suggested that the symbiotic relationships with six different *Glomus* species (*G mosseae, G. etunicatum, G. microaggregatum, G. geosporum, G. claroideum*, and *R. irregularis*) favored the ability of the olive root to expand in a larger volume of soil and to regulate stomatal conductance and root hydraulic conductivity, thus improving the photosynthetic activities and water relation status of the young olive plants. The significantly more depleted δ¹³C values in treated trees indicated elevated intercellular CO_2_ concentrations (C_i_/C_a_). Since treated trees also displayed higher growth rates and greater foliar expansion, the elevated C_i_/C_a_ most likely reflects enhanced stomatal conductance, allowing higher rates of CO_2_ assimilation (Battipaglia and Cherubini, 2022). The reduced interannual variation in δ¹³C of treated trees (SD = 1.3 versus 1.8 for controls) indicated more stable photosynthetic performance under the observed seasonal temperature variability (**Figure 2**).

The overall depletion of δ¹^5^N observed in biostimulant-treated trees is consistent with a microbial-mediated nitrogen (N) uptake. While overall wood N% remain similar, the isotopic composition reveals information about which N pools and acquisition mechanisms are actually being exploited.

AMF preferentially transfer N to host plants through hyphal networks that extend into soil micropores inaccessible to uncolonized roots. This hyphal N uptake involves selective assimilation and transfer of organic N compounds (amino acids, peptides, oligosaccharides containing N), which undergo different isotopic fractionation than the high-affinity inorganic N transporters dominating in non-mycorrhizal roots (Duan et al., 2024). The resulting N in root-mycorrhizal plant tissues carries the isotopic signature of microbial N transformations, typically characterized by δ^15^N depletion relative to bulk soil N (Hawkins and George, 1999; Hobbie and Colpaert, 2003; Jin et al., 2005; He et al., 2009; Hobbie and Högberg, 2012; Keller and Phillips, 2019). Such a pattern has been more widely and consistently observed in ectomycorrhizal symbionts, which can reduce δ¹^5^N values by up to −12‰ (Evans, 2001). However, the extent and direction of ¹^5^N partitioning between AMF and hosts vary among plant species (Courty et al., 2015) and mycorrhizal types (Gomes et al., 2023). Recent findings show that both intraradical and extraradical AMF hyphae are generally δ¹^5^N-enriched relative to the leaves and roots of their host plants, including grasses and legumes (Klink et al., 2020) as well as trees (Klink et al., 2022).

Furthermore, mycorrhizal fungi and rhizosphere microorganisms themselves induce isotopic fractionation during N acquisition and transfer to the host plant, often resulting in lower δ¹^5^N values in plant tissues compared with non-mycorrhizal plants (Gebauer and Meyer, 2003). Thus, the depleted δ¹^5^N signatures detected in xylem tissues of treated trees likely reflect an increased contribution of microbially mediated N pathways rather than changes in bulk N availability (**Figure 5**).

The seasonal pattern in δ^15^N values within individual rings provides evidence of dynamic shifts in N-acquisition pathways throughout the growing season. In treated trees, the marked δ^15^N minimum during mid-season likely may reflect peak mycorrhizal fungal activity and maximum reliance on microbially-mediated N assimilation, potentially driven by intermediate soil temperatures and C availability from photosynthetic exudates. The subsequent δ^15^N enrichment in late-season wood suggests a shift toward reduced mycorrhizal activity as soil temperature declines (**Figure 2**).

However, given the limited sample size, these interpretations should be considered preliminary and warrant confirmation through further studies involving larger experimental populations and direct assessments of belowground microbial activity. The significant correlation between C% and δ^15^N suggests a coordinated shift in C and N metabolism. While the overall C% of the wood matrix decreases, the contribution of microbially-mediated N acquisition to total plant N increases, manifesting lower δ^15^N. The absence of correlation between δ^15^N and absolute N% suggests that the MICOSAT F^®^ biostimulant does not operate by increasing absolute N availability, but rather by reorienting N acquisition toward microbially-mediated pathways. Unlike conventional fertilization, absolute xylem N% remains stable, while the source of N has shifted toward more metabolically available forms (amino acids from mycorrhizal partners, organic N from fungal-supplied compounds) that are more readily assimilated and utilized in supporting photosynthetic performance.

These interpretations, however, should be considered preliminary, as they rest on indirect evidence and would require confirmation through direct assessment of AMF root colonization, NSC dynamics, and the seasonal turnover of belowground microbial communities.

## 5 Conclusion

This study provides novel ecophysiological insights into how microbial biostimulants modulate growth, carbon (C) assimilation and nitrogen (N) acquisition in young olive trees. The application of MICOSAT F^®^ consortium significantly enhanced vegetative growth and biomass accumulation in Leccino cultivar over a two-year period, demonstrating the agronomic potential of microbial biostimulants in olive cultivation.

The integration of dendrochronological approaches with stable isotope analyses revealed coordinated metabolic shifts associated with biostimulant treatment. The reduction in stem wood C% coupled with enhanced growth suggests a reallocation of photosynthates toward non-structural carbohydrates and belowground symbiotic partnerships, representing a C investment in the mycorrhizal network consistent with the higher AMF propagule density observed in the rhizosphere of treated trees. This symbiotic cost is balanced by substantial benefits: enhanced stomatal conductance and photosynthetic capacity, as evidenced by depleted δ¹³C values, and improved access to soil N pools through microbially-mediated pathways, as indicated by depleted δ¹^5^N signatures. Treated trees showed reduced interannual variation in δ¹³C, suggesting greater physiological stability under seasonal temperature variability typical of Mediterranean environments. The shift toward microbially-mediated N acquisition, without increasing absolute N content, indicates improved nitrogen-use efficiency through qualitative rather than quantitative changes in nutrient acquisition.

These findings highlight the potential of MICOSAT F^®^ biostimulant as a sustainable tool for enhancing olive tree performance and for supporting physiological mechanisms that may contribute to adaptation in Mediterranean agroecosystems. While the controlled environment of this study allowed the isolation of biostimulant-driven responses, the observed seasonal patterns primarily reflect temperature-driven variability rather than water-limited conditions. In contrast, the more complex and heterogeneous environment of olive groves may modulate plant-microbe interactions, influencing both the magnitude and stability of these effects. Future research should therefore investigate the long-term effects of biostimulant application under realistic agronomic conditions and evaluate plant performance under specific abiotic stressors, such as drought and salinity, to more directly assess the adaptive benefits of these plant-microbe partnerships.

## Data Availability

The raw data supporting the conclusions of this article will be made available by the authors, without undue reservation.
